# A novel feature ranking method for prediction of cancer stages using proteomics data

**DOI:** 10.1371/journal.pone.0184203

**Published:** 2017-09-21

**Authors:** Ehsan Saghapour, Saeed Kermani, Mohammadreza Sehhati

**Affiliations:** Department of Biomedical Engineering, School of Advanced Technologies in Medicine, Isfahan University of Medical Sciences, Isfahan, Iran; Southwest University, CHINA

## Abstract

Proteomic analysis of cancers' stages has provided new opportunities for the development of novel, highly sensitive diagnostic tools which helps early detection of cancer. This paper introduces a new feature ranking approach called FRMT. FRMT is based on the Technique for Order of Preference by Similarity to Ideal Solution method (TOPSIS) which select the most discriminative proteins from proteomics data for cancer staging. In this approach, outcomes of 10 feature selection techniques were combined by TOPSIS method, to select the final discriminative proteins from seven different proteomic databases of protein expression profiles. In the proposed workflow, feature selection methods and protein expressions have been considered as criteria and alternatives in TOPSIS, respectively. The proposed method is tested on seven various classifier models in a 10-fold cross validation procedure that repeated 30 times on the seven cancer datasets. The obtained results proved the higher stability and superior classification performance of method in comparison with other methods, and it is less sensitive to the applied classifier. Moreover, the final introduced proteins are informative and have the potential for application in the real medical practice.

## Introduction

Cancer has always been one of the most fundamental health problems of the human society. Every year, between 100 and 350 out of every 100,000 people die due to cancer in the worldwide [1–4]. Understanding the nature of cancer, which caused by the malfunction of the mechanisms that regulate growth and cell division, has always been a topic of interest to researchers. The development of molecular biology in recent decades enhanced understanding of complex interactions of the genetic variants, transcription and translation [5]. Proteomic studies can play a critical role in prevention, early detection and treatment of cancer. Given that proteomic studies can help identify cancer biomarkers, it might cause early detection and treatment of cancer [6, 7].

The robustness of microarray-derived cancer biomarkers that have been identified by using gene expression profiles is very poor [8, 9]. Thus, the evaluation of tumor cells at protein expression levels, which are more robust than gene expression level, is necessary to explain causes of tumor proliferation. And it will help us to find potential drug targets and to illustrate off-target effects in cancer medicine [10].

Zhang et al. [10] utilized the protein expression profiles for classifying ten types of cancers. They applied minimum redundancy maximum relevancy (mRMR) and incremental feature selection (IFS) methods for selecting 23 out of 187 proteins on the protein array, which used as the inputs of sequential minimal optimization (SMO) classifier. Sonntag et al. [11] have introduced a novel biomarker selection workflow to extract four discriminative biomarkers from reverse phase protein array (RPPA) data on luminal breast cancer.

Kaddi and Wang [12] employed three different approaches for feature selection (two filter and one wrapper methods) and six methods for classification (four individual binary and two ensemble classification methods) to predict early stage of cancer in Head and Neck Squamous Cell Carcinoma using proteomic and transcriptomic data.

Stafford et al. [13] randomly generated two libraries, each of them contained approximately 10000 peptide sequences, then they used ANOVA and t-test for feature selection and the linear discriminant analysis (LDA), naive bayes (NB) and support vector machine (SVM) for classification. Numerous studies have been reported for identification of biomarkers that influence in the early detection of ovarian cancer [14].

Nguyen et al. [15, 16] presented a novel feature selection method by integrating the five filter-based feature selection approaches (i.e., t-test, ROC, Wilcoxon, Entropy, and SNR) through an analytic hierarchy process (AHP). AHP, which is a multi-criteria decision analysis method, is used for classification of normal and tumor tissue by means of different classifier algorithms (i.e. Interval type-2 Fuzzy Logic (FL) [17], Hidden markov model (HMM) [18], k-nearest neighbors (kNN) [19], support vector machine (SVM) [20], etc.).

In this research, we proposed a hybrid model for prediction of cancer stages using RPPA data. The novelty of the proposed method relies on the feature ranking using TOPSIS. To improve the stability and accuracy of the final extracted biomarkers, we modified the feature selection workflow and utilized the best classification model among the well-known seven classifiers (i.e. SVM [20], Random Forest (RF) [21], Decision Tree (DT) [22], LDA [23], NB [24], FL [25], and kNN [19]).

As demonstrated by a series of recent publications [26–32], and in agreement with the famous 5-step rule [33], we should comply with the following five step instruction to construct a really useful prediction method for a biomedical system; (1) select or construct a valid benchmark dataset to train and test the predictor model, (2) formulate the statistical samples with an effective mathematical expression that can truly reflect their intrinsic correlation with the target to be predicted; (3) develop or introduce a powerful algorithm to run the prediction, (4) properly perform cross-validation tests to objectively evaluate the anticipated accuracy of the predictor, (5) establish a user-friendly and publicly accessible web-server for the predictor.

The rest of the paper is organized as follows: In Section 2, the utilized database is introduced and a detailed description of the proposed protein ranking and classification methods is presented. In Section 3, the evaluation results of the various protein selection methods in combination with the various kinds of classifiers are described. The related issues of cancer classification are discussed in Section 4. We conclude the paper in Section 5.

## Materials and methods

### Dataset

Proteomic data, including 2101 patient samples from 7 cancer types were downloaded from The Cancer Proteome Atlas (TCPA) [34]. For each sample, the expressions of 187 proteins were taken by RPPA. We used RPPA, an antibody-based high-throughput technique, for analyzing concurrent expression levels of hundreds of proteins in a single experiment.

The related pathological information for each patient in the TCPA dataset was downloaded from Broad Institute TCGA (https://confluence.broadinstitute.org/display/GDAC/Dashboard-Stddata and http://tcpaportal.org/tcpa/download.html). Then, we divided the samples into two groups of early stage (stage I and II) and advanced stage (stage III and IV).

In TCPA, the proteins are divided into three groups including "validated", "under evaluation" and "used with caution". In this work, we only used 115 validated labeled proteins per patient to obtain reliable results. See Table 1 for details.

**Table 1:** Summary of the utilized cancer datasets.

Cancer	# early-stage	# advanced-stage	# Total
READ	60	62	122
HSNE	48	152	200
LUSC	158	35	193
COAD	187	139	326
OV	33	370	403
UCEC	321	83	404
KIRC	263	190	453
Total Number of Samples	1070	1031	2101

READ: Rectum adenocarcinoma, HSNE: Head and Neck sequamous cell carcinoma, LUSC: Lung sequamous cell carcinoma, COAD: Colon adenocarcinoma, OV: Ovarian serous cystadenocarcinoma, UCEC: Uterine Corpus Endometrioid Carcinoma. KIRC: Kideny renal clear cell carcinoma.

We used the R software and FEAST Toolbox [35] in MATLAB to implement different classification models and feature selection algorithms. All the cleaned data and the algorithms scripts used in this manuscript, can be downloaded from www.github.com/E-Saghapour/FRMT.

### Hybrid models

**Fig 1:**
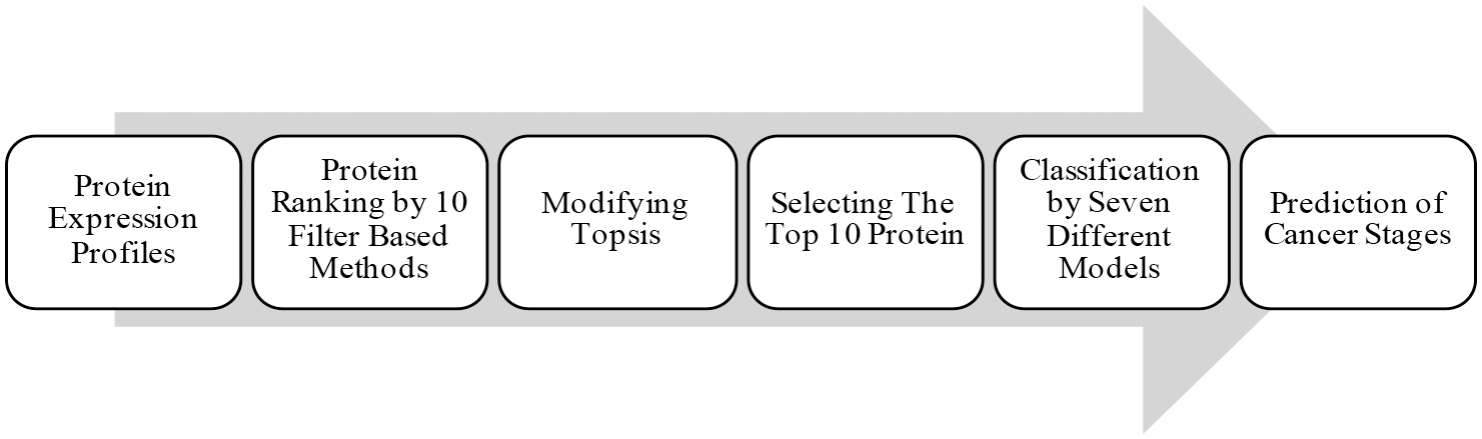
Schematic of the proposed data analysis procedure. The whole procedure from processing the protein expression profiles to prediction of the cancer stages is illustrated.

The hybrid model approaches were used by many previous investigators to study various biological or biomedical problems [36–40]. The stratification of cancer can be considered as traditional pattern recognition problems. Data analysis procedure, including feature selection and classification steps, is shown in Fig 1. The explanation of different blocks in Fig 1 is presented in the next subsections.

#### Feature selection

**Fig 2:**
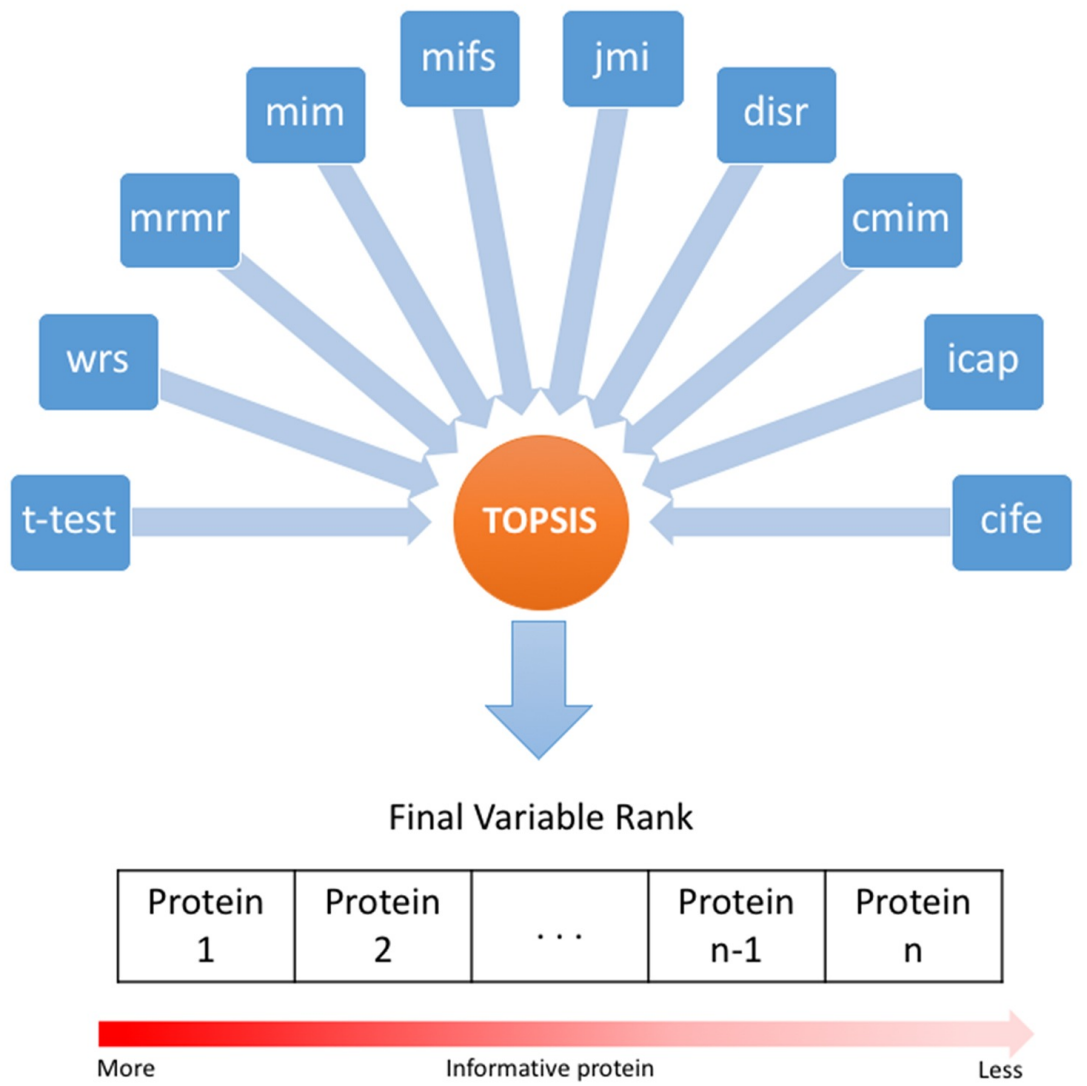
Feature ranking procedure. TOPSIS is used for integration of different FFS methods for proteins ranking.

In a filter feature selection (FFS) method, a criterion function would be used for independently ranking features. Then, the top ranked features, called informative features, would be used in the classification model. Various criterion functions have been introduced and applied to the gene expression profiles that led to different subset of genes with different classification performance. Although the FFS methods produce unstable results in different datasets, but they are robust against overfitting. FFS methods can also be applied to the protein expression profiles for protein ranking, however, they do not take into account protein-protein interactions. In this study, a novel ensemble method is proposed to improve the stability of results obtained by integrating common FFS methods (Table 2). We utilized the TOPSIS method to score the proteins and choose the most informative ones for classification (Fig 2). The TOPSIS method is described in detail in the next section.

**Table 2:** Common filter-based feature selection methods.

Criterion	Full name
*t-test*	Two sample t-test
*wrs*	Wilcoxon rank sum
*mrmr*	Max-Relevance Min-Redundancy
*mim*	Mutual Information Maximization
*mifs*	Mutual Information Feature Selection
*jmi*	Joint Mutual Information
*disr*	Double Input Symmetrical Relevance
*cmim*	Conditional Mutual Info Maximization
*icap*	Interaction Capping
*cife*	Conditional Infomax Feature Extraction

#### TOPSIS method

The TOPSIS was first presented by Hwang and Yoon in 1981 [41]. It is a multi-criteria decision analysis method relied on selecting the option that its geometric distances from the positive ideal solution (PIS) and the negative ideal solution (NIS) are the shortest and longest, respectively.

The workflow of the TOPSIS method contains the following seven steps:

1. Generating an *m*-by-*n* evaluation matrix contains *m* alternatives $A_{1},A_{2},\ldots ,A_{m}$, each assessed by *n* local criteria $C_{1},C_{2},\ldots ,C_{n}$.
2. Normalizing the decision matrix:
  $$u_{ij}\;=\frac{x_{ij}}{\sqrt{\sum_{k=1}^{m}x_{kj}^{2}}};\;i=1,\ldots ,m;\;j=1,\ldots .,n.$$ (1)
  Where *x*_*ij*_ is the score of alternative *A*_*i*_ with respect to the criterion *C*_*j*_.
3. Calculating the weighted normalized decision matrix which its values *V*_*ij*_ are computed as:
  $$V_{ij}=W_{i}\times u_{ij};\;j=1,2,\ldots ,m;\;i=1,2,\ldots ,n.$$
  let *W*_*i*_ = [*w*_1_, *w*_2_,…,*w*_*n*_] be the vector of local criteria weights satisfying $\sum_{i=1}^{n}W_{i}\;=\;1$.
4. Determining the positive ideal (*A*^*+*^) and negative ideal (*A*^*-*^) solutions as follows:
  $$A^{+}=\left\{v_{1}^{+},\ldots ,\;v_{n}^{+}\right\}=\left\{\left(\underset{i}{\text{max}}V_{ij}|\;j\in J\right),\;\left(\underset{i}{\text{min}}V_{ij}|\;j\in J\prime \right)\right\}.$$ (2)
  $$A^{-}=\left\{v_{1}^{-},\ldots ,v_{n}^{-}\right\}=\left\{\left(\underset{i}{\text{min}}V_{ij}|\;j\in J\right),\left(\underset{i}{\text{max}}V_{ij}|\;j\in J\prime \;\right)\right\}.$$ (3)
  J={j=1,2,3,…,n|jassociatedwithbenefitcriteria}. (4)
  J′={j=1,2,3,…,n|jassociatedwithcostcriteria}. (5)
  In the proposed method, all criteria are considered as benefit, therefor *J’* is empty and (2), (3) will be reduced to (6), (7);
  $$A^{+}=\left\{v_{1}^{+},\ldots ,\;v_{n}^{+}\right\}=\left\{\underset{i}{\text{max}}V_{ij}|j\in J\right\}.$$ (6)
  $$A^{-}=\left\{v_{1}^{-},\ldots ,\;v_{n}^{-}\right\}=\left\{\underset{i}{\text{min}}V_{ij}|j\in J\right\}.$$ (7)
5. Measuring the Euclidean Distances between each alternative and both the positive and negative ideal, which are calculated as follows:
  $$P_{i}^{+}=\sqrt{\sum_{j=1}^{n}{\left(v_{ij}-v_{j}^{+}\right)}^{2}};\;i=1,2,\ldots ,m.$$ (8)
  $$P_{i}^{-}=\sqrt{\sum_{j=1}^{n}{\left(v_{ij}-v_{j}^{-}\right)}^{2}};\;i=1,2,\ldots ,m.$$ (9)
6. Computing the relative closeness to the ideal solution by Eq (10).
  $$H_{i}=\frac{P_{i}^{-}}{P_{i}^{+}+P_{i}^{-}}\;;\;i=1,2,\ldots .,m;\;0\leq \;H_{i}\;\leq 1.$$ (10)
7. Ranking alternatives based on the H value of each parameter. *H*_*i*_ = 1 indicates the highest rank and *H*_*i*_ = 0 indicates the lowest rank.

**Fig 3:**
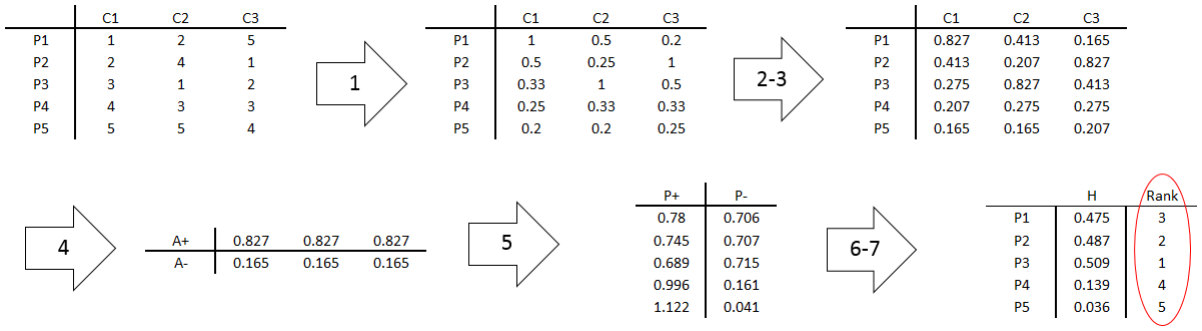
Illustration of TOPSIS. This illustrative example explains the functionality of TOPSIS method in a simple application in which we have 5 alternatives, P1-P5, and 3 criteria, C1-C3.

Fig 3 illustrates the whole procedure of TOPSIS method for a simple example in which we have 5 alternatives (proteins expression), P1-P5, and 3 criteria (methods of feature selection), C1-C3. Moreover, we considered equal weights for all feature selection methods (criteria).

#### Classification

In this study, we utilized seven models for classification including SVM, RF, DT, LDA, NB, FL, and kNN.

SVM rely on the concept of decision planes that specify decision borders. Classification task performed by building hyperplanes in a multidimensional space that distinct various class labels. The classes that have nonlinear boundaries in the input space employ the kernel function method to map the input space in to a higher dimensional feature space in which linear differentiation may be feasible. The kernel trick computes all training data without using or knowing the mapping, thus high dimensionality of the feature space does not increase computational cost of classification and training task.

RF is an ensemble classifier comprised of many decision trees. The mode of class output obtained by individual trees would be the class that is output by RF[42]. The Random Decision Forests learning algorithm was developed by Leo Breiman [21] based on decision trees, which are non-parametric supervised learning approaches used for regression and classification. Using of a set of tree classifiers and randomness in the RF design led to good accuracy and stability of the resulting classifier.

RF is a classifier including a set of tree-structured classifiers {*g*(*x*, *b*_*k*_) *k* = 1, 2,…}, where the {*b*_*k*_} are independent identically distributed random vectors and each tree puts a unit vote for the famous class at input *x*. The RF method (along with other ensemble learning methods) has been very popular in biomedical research, and it considers random tree building using both bagging and random variable selection [43].

Fuzzy Inference System (FIS) is a method of mapping the input space to an output space using FL. FIS attempts to formalize the reasoning procedure of human language by means of FL and building fuzzy IF-THEN rules. The procedure of fuzzy inference involves all of the sections that are explained in Membership Functions, Logical Operations, and If-Then Rules. They have become strong methods to afford various problems such as uncertainty, imprecision, and non-linearity. They are generally used for identification, classification, and regression works. Instead of employing crisp sets as in classical rules, fuzzy rules exploit fuzzy sets. Rules were initially taken from human experts through knowledge engineering procedures. However, this approach may not be possible when facing complicated tasks or when human experts are not accessible [44].

The kNN algorithm, one of the popular machine learning algorithms, is a non-parametric method used for classification and regression predictive problems. In both cases, the input vector contains the k closest training samples in the feature space. The output is dependent to value of k whether it is used for classification or regression. In kNN classification, the output is a class membership. An object is classified by a majority vote of its neighbors with the object being allocated to the class most usual between its k nearest neighbors. The best election of k depends on the data; a good k can be elected by different heuristic methods. Larger values of k decrease the effect of noise on the classification, but it creates boundaries between classes less distinct. A drawback of the kNN algorithm is its sensitivity to the local structure of the data. In kNN regression, the output is the property value for the object. This value is the average of the values of its k nearest neighbors rather than voting from nearest neighbors [45].

LDA is a method used in pattern recognition, statistics, and machine learning to detect a linear combination of features that separate two or more classes of objects and is an extension of Fisher's linear discriminant; Such combination might be used as a linear classifier, or, more generally, for dimensionality reduction before later classification. LDA attempts to represent one dependent variable as a linear combination of other features and is closely relevant to analysis of variance and regression analysis [46]. LDA is closely relevant to factor analysis and principal component analysis (PCA) in that they both look for linear combinations of variables which best illustrate the data. LDA clearly efforts to model the diversity among the classes of data. PCA, on the other hand, does not take into account any diversity in class, and factor analysis creates the feature combinations according to the differences rather than similarities [46].

The Bayesian Classification is a statistical method for classification that illustrates a supervised learning strategy. Bayesian classification provides practical learning algorithms in which the former knowledge and the observed data can be combined. Bayesian Classification provides an effective perspective for evaluating and understanding many learning algorithms. It is not affected by noise in input data and calculates clear probabilities for hypothesis. The NB classifier is used when features are independent of each other within each class, but it works well in practice even when that independence assumption is not valid. NB classifier requires a small amount of training data to estimate the parameters such as mean and variance of the variables necessary for classification [47].

### Performance measures

K-fold cross-validation test, independent dataset test, sub-sampling test and jackknife cross-validation test are four widely used classes of schemes in statistical classify for examining the performance of a prediction model [48–53]. The jackknife test has been widely used in Bioinformatics [54–68], because it can achieve unique outcome [33, 69]. However, it is time-consuming. For saving the computational time, in this study, ten-fold cross-validation was used to investigate the performance of the prediction model. In k-fold cross-validation, the data is divided into k subset, each time, one of the k subsets and k-1 subsets are used as test and train data, respectively. Then the mean error across all k experiment is calculated. Since the utilized dataset is unbalanced in terms of number of samples in two groups of early and advanced stages, the Area under Curve (AUC) and Matthews Correlation Coefficient (MCC) were used.

The MCC, introduced by Brian W. Matthews [70], is used for measuring the quality of binary classification. The MCC is a number between -1 and +1. Values of 1 and 0 demonstrate a perfect and random prediction, respectively. In addition, -1 represents total disagreement between the predicted and actual values. It can be computed from the confusion matrix as:

$$MCC=\frac{\left(TP\times TN\right)-\left(FP\times FN\right)}{\sqrt{\left(TP+FP\right)\left(TP+FN\right)\left(TN+FP\right)\left(TN+FN\right)}}.$$ (11)

where TP is the number of true positives (early stage), TN is the number of true negatives (advanced stage), FP is the number of false positives, and FN is the number of false negatives. Eq 11 can be represented in another form like Eq. 11 in [40], which were derived by Xu et al. [29] and Lin et al. [40] based on the symbols introduced by Chou in studying signal peptides and those used in many recent studies [26–32]. The set of metrics is valid only for the single-label systems. For the multi-label systems which has become more frequent in systems biology [71] and systems medicine [37, 72–74], a completely different set of metrics as defined in [75] is needed.

Moreover, the AUC is defined as the area under the ROC curve, which illustrates the performance of a binary classifier system as its discrimination threshold is varied. An AUC of 1, 0.5, and under 0.5 indicates a perfect, random, and bad classifier, respectively.

## Results

As can be seen from the data in Table 1, the KIRC cancer with 453 samples, contains the most samples in the whole dataset. The UCEC Cancer is the second-most with 404 samples. According to the pathologic stage, data are unbalanced and the READ and OV data has the less and the most unbalanced level, respectively.

**Fig 4:**
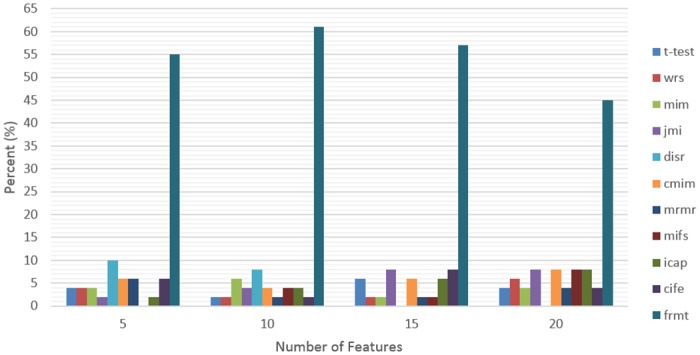
Effect of feature subset size on performance. The winning frequency is calculated for different feature selection methods for various sizes of feature subsets.

To present the performance of the proposed FRMT method, we have provided 7 tables; one table for each cancer data (S1 Table), which demonstrated the effect of applying different feature selection technique on various classifier architectures. In this regard, MCC and AUC are used as evaluation measures in 30 repetitions of 10-fold cross-validation procedure. For a fair performance evaluation, we should consider different constraints that affect the classification performance such as: train dataset, classifier model, and the number of selected features. In this regard, we should evaluate different possible combinations, which contains 49 states due to the seven classifiers that applied on seven datasets. Then, we select subset of features with different sizes (5, 10, 15 and 20) obtained by each feature selection method, considering which method reaches the highest accuracy in each of 49 state. Fig 4 shows the percentage of states that each feature selection method reached the best performance (winning frequency) for various numbers of features. As it is shown in Fig 4, the proposed method has reached the best result for all sizes of feature subsets in comparison with other methods, and the peak of result obtained by using 10 features.

**Fig 5:**
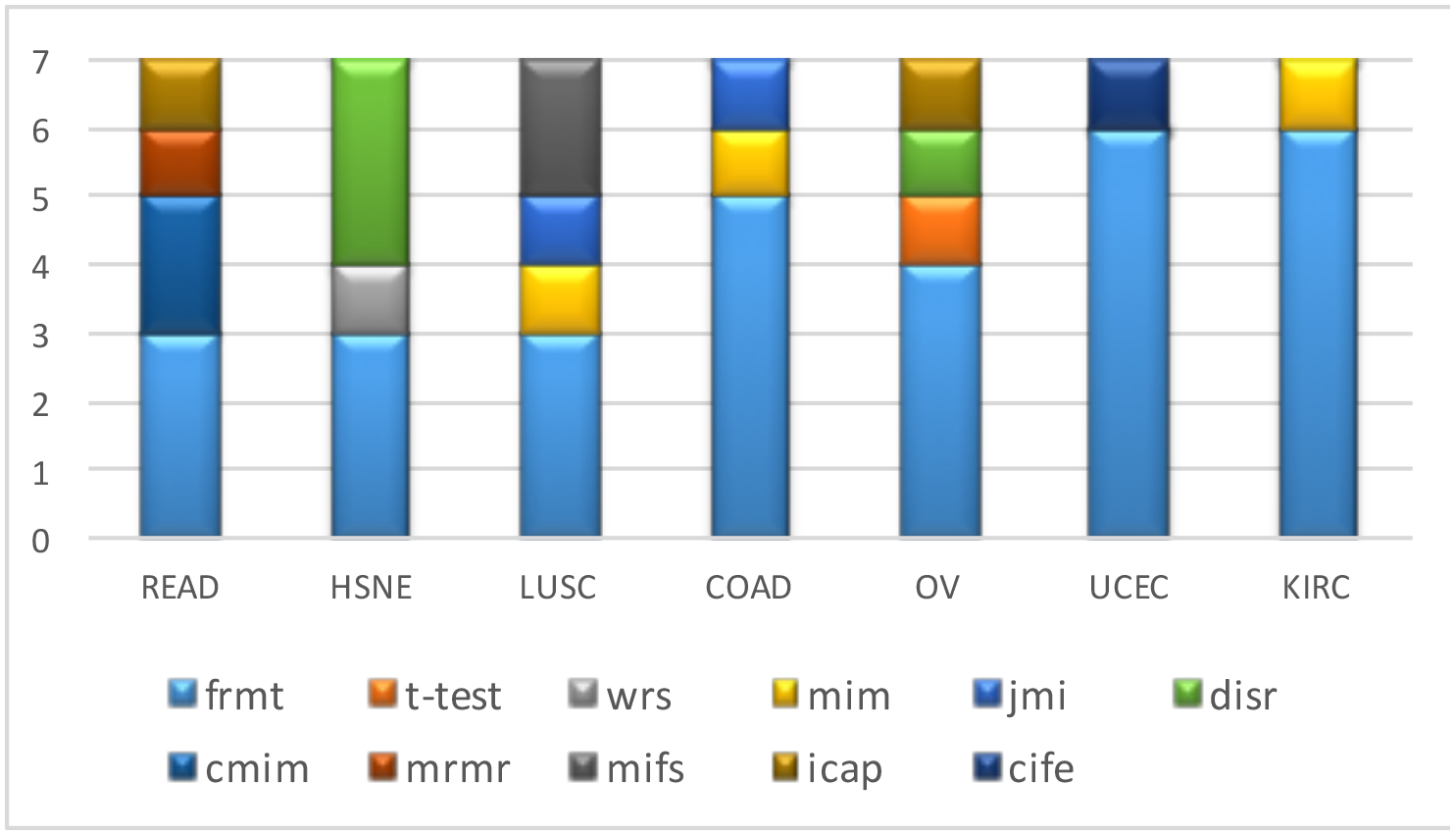
Comparison of FFS methods in different datasets. The winning frequency is calculated for different feature selection methods in each dataset, regarding the classification performance.

After this point the same number of features (top 10 proteins) has been selected as the input of all classifiers in all experiments. The best results in the tables are highlighted by shading. The frequency of selection of each feature selection method as the best, or winning frequency regarding the classification performance, is depicted in Fig 5. For each cancer, each classifier model obtained the best answer with only one of the eleven feature selection methods.

**Fig 6:**
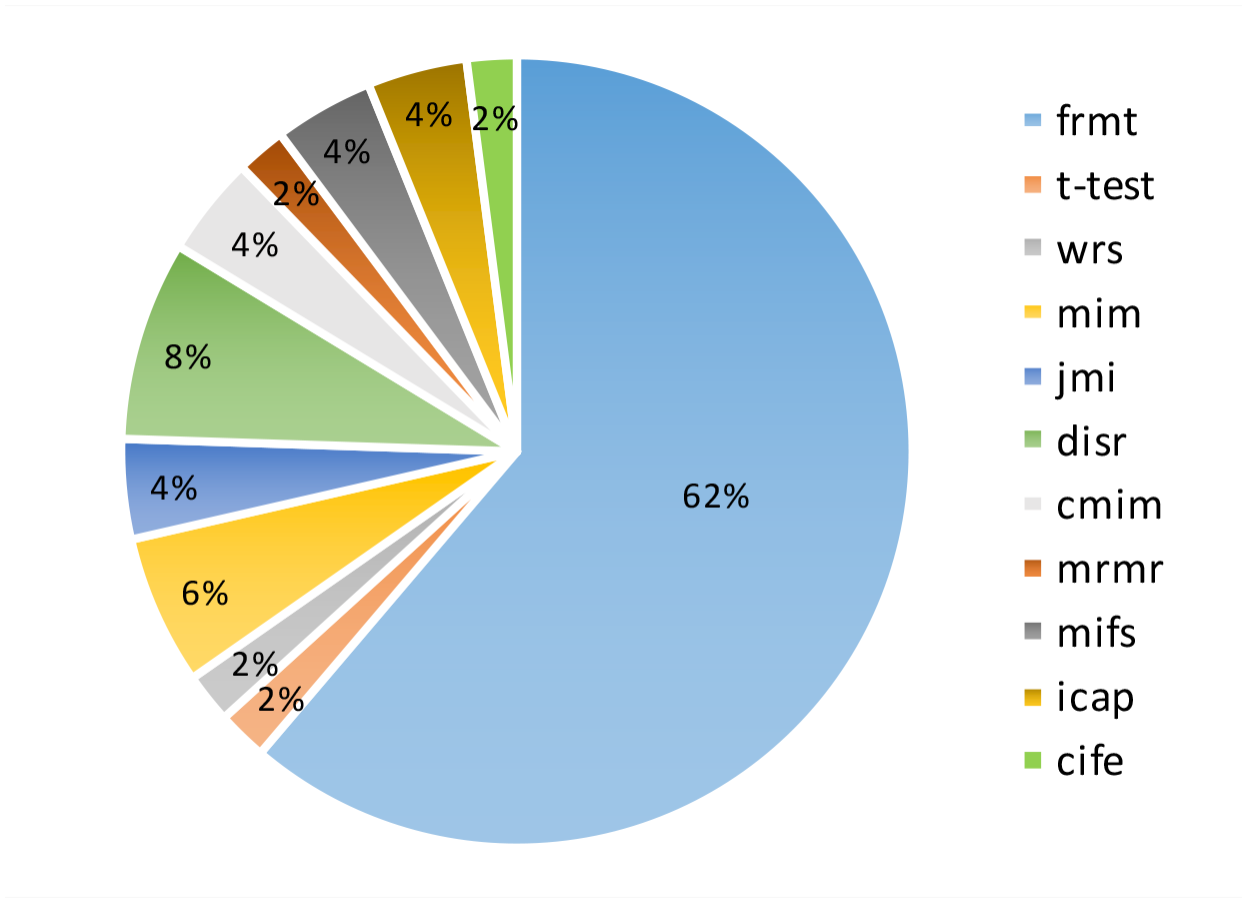
Final comparison of different FFS methods. The winning frequency of all feature selection methods is illustrated in a pie chart for all datasets.

Fig 6 illustrates the winning frequency of all feature selection methods in the whole dataset. The method with larger segment on the pie chart demonstrates the better approach.

The results presented in S1 Table are summarized in Table 3. The left part of Table 3 demonstrates a comparative analysis of the FRMT method performance by applying different classification models in seven datasets. In the right part of Table 3, the best results of every feature selection method in combination with a classifier that led to the best performance for prediction of cancer stage are shown.

**Table 3:** Comparison of the FRMT method with other methods for whole cancers.

	FRMT method			Other methods			
Cancer	MCC	AUC	Classifier	MCC	AUC	Criteria	Classifier
READ	**0.23±0.0025**	**61.59±0.61**	LDA	**0.23±0.0021**	59.09±0.31	*cmim*	SVM
HSNE	**0.37±0.0002**	**69.55±0.07**	NB	0.27±0.0018	60.59±0.31	*disr*	KNN
LUSC	**0.27±0.002**	**59.02±0.28**	KNN	0.16±0.0068	57.93±1.69	*mim*	DT
COAD	**0.18±0.0015**	**58.55±0.31**	SVM	0.15±0.0013	56.97±0.3	*mim*	SVM
OV	**0.27±0.0007**	**61.57±0.11**	NB	0.17±0.0013	54.58±0.12	*cife*	NB
UCEC	**0.26±0.0011**	**56.54±0.08**	NB	0.16±0.0005	55.96±0.08	*disr*	NB
KIRC	**0.34±0.0001**	**66.54±0.03**	NB	0.28±0.0005	63.51±0.13	*wrs*	RF

After applying the FRMT method in different datasets, top ranked proteins were extracted and the name of first 10 informative ones were reported in S1 Table.

## Discussion

In this study, a new approach called FRMT method was proposed to select protein biomarkers. 10 FFS methods were integrated to extract the best stage prediction cancer biomarkers. Finding the best proteins via a multi-criteria decision analysis method, the FRMT method demonstrates a proficient method for ranking proteins using protein expression profile data without concerning about the selection of suitable FFS method for a specific problem.

The performance of six well-known classifiers was evaluated and reported using 10 top ranked proteins selected by the FRMT and other FFS approaches. The results indicate that the FRMT method is more advantageous than other FFS methods in terms of robustness in classification performance; By measuring the number of times that a method obtained the best results, we observed that the best frequency has been achieved by the FRMT method with 6 out of 7 times in UCEC and KIRC cancer (Fig 5). Furthermore, in the READ and LUSC cancer dataset, the maximum frequency of 3 out of 7 has been reached by the FRMT method. In the HSNE cancer, the frequency for *disr* and FRMT methods are equal to 3. It should be noted that some methods of feature selection were never chosen as the best model.

Looking at the pie chart in Fig 6, in 62 percent of experiments the FRMT method achieved the best classification performance in the whole proteomic dataset. Afterward, the *disr* and *mim* methods reached to success rate of 8 and 6 percent, respectively.

As it is reported in Table 3, the best performance in prediction of cancer stage evaluated by using AUC and MCC has occurred in HSNE dataset. The AUC of 69.55 with SE (Standard Error) of 0.07 and MCC of 0.37 with SE of 0.0002 are the best results among all dataset achieved by the FRMT method as feature selection and NB method as classifier. It should be noted that in HSNE dataset, the *disr* method reached the second-best place by AUC of 60.59 with SE of 0.31 and MCC of 0.27 with SE of 0.0018.

Comparison of the FRMT method with other methods in Table 3 are suggestive of the *wrs* method achieving the best results among other FFS methods; AUC of 63.51 with SE of 0.13 and MCC of 0.28 with SE of 0.0005 have been achieved by using RF as classifier in KIRC dataset. However, in the KIRC dataset, the FRMT method already obtained the best result with NB classifier, which are AUC of 66.54 with SE of 0.03 and MCC of 0.34 with SE of 0.0001.

As it is seen from the data in Table 3, the NB classifier was achieved the best results in the majority of experiments evaluated various feature selection methods. NB achieved the best performance in 4 out of 7 datasets using the proposed method, and in two datasets by applying *cife* and *disr* methods. Notably, the SVM classifier obtained the second place.

**Fig 7:**
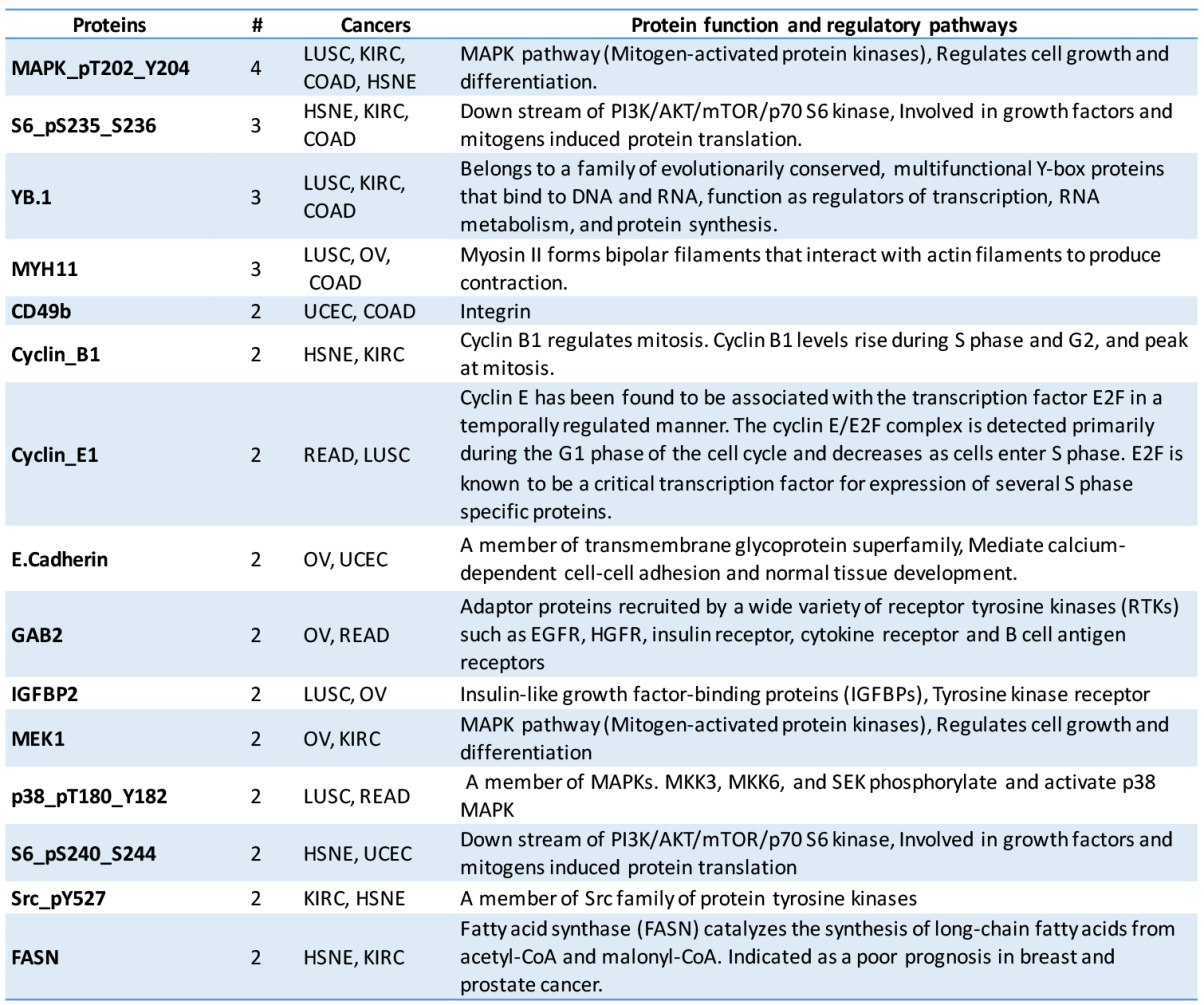
Detail description of top ranked proteins. The name, frequency of selection, related cancer, function and regulatory pathways of informative proteins are reported, which are appeared more than one time in whole cancers among the 10 top ranking of selected proteins by FRMT method.

Top-ranked protein selected by FRMT method from each dataset, showed significant overlap with recently discovered biomarkers that were associated with cancer development. According to Fig 7, MAPK_pT202_Y204 is the most frequently selected protein from 4 datasets among top 10 ones by FRMT. The striking point about the MAPK_pT202_Y204 is its significant role in MAPK pathway (Mitogen-activated protein kinases) and regulation of cell growth and differentiation [34].

In addition, S6_pS235_S236 which involved in growth factors and mitogens induced protein translation [34], is the second frequently selected protein selected from 3 datasets among top 10 ones by FRMT.

Gab2 that is selected by FRMT as the most informative protein in the READ dataset is recently introduced as an overexpressed protein in several cancer types [76–78]. Moreover, several researchers have reported that overexpression of Gab2 stimulates cell proliferation, cell transformation, and tumor progression; Ding et al. [79] showed Gab2 overexpression in clinical colorectal cancer (CRC) specimens. Moreover, Gab2 is selected by FRMT as the second discriminative protein in OV dataset, and this is in concordance with recent studies that reported Gab2 amplification and overexpression in a subset of primary high-grade serous ovarian cancers and cell lines [78]. Furthermore, the expression level of IRS1, which is selected by FRMT as the second discriminative protein in READ dataset, was utilized by Hanyuda et al. as a predictive marker for classification of patients according to their survival benefit gained by the exercise [80].

About S6 phosphorylation(S6_pS240_S244), which is selected by FRMT as the most discriminative protein in the HSNE dataset, previous studies have revealed its high occurrence in HNSCC specimens and demonstrated its correlation on clinical outcomes [81].

Bcl-2 protein is chosen by FRMT as an important marker in the COAD dataset; This finding broadly supports the work of Poincloux et al., linking loss of Bcl-2 protein expression with increase in relapse of stage II colon cancer, and it could be a potential histo-prognostic marker in therapy decision making [82].

Many studies [53, 60–62, 83–85] have demonstrated that high dimension data will bring about information redundancy or noise that results in bad prediction accuracy, over-fitting that results in low generalization ability of prediction model, and dimension disaster which in turn is a handicap for the computation. Thus, a novel two-step feature selection technique was applied to optimize features.

As demonstrated in a series of recent publications (see, e.g., [26–32, 86, 87]) in evaluating new prediction/classification methods, user-friendly and publicly accessible web-servers will significantly enhance their impacts [88, 89], we shall try to provide a web-server in our future work for online application of the method presented in this paper. Moreover, for extending our experiment, we shall consider combining different feature selector as in [90].

## Conclusion

Various FFS methods may lead to diverse biomarkers with different discriminative power in different datasets. However, the proposed FRMT method can help researchers to select more stable biomarkers from protein expression profiles by integrating various FFS methods. The proposed method has the advantage of stability and classification performance compared with other approaches. However, it suffers from the computational complexity problem comparing to FFS methods. On the other hand, the FRMT method in comparison to the wrapper feature selection approaches, has lower computational complexity and produce more general results without overfitting.

## Supporting information

S1 Table Name of proteins and their classification performance in all datasets. The results obtained for 10 formative biomarkers selected by FRMT and reported in different sheets for each dataset.
(XLSX)
